# European Society of Organ Transplantation (ESOT) Consensus Report on Downstaging, Bridging and Immunotherapy in Liver Transplantation for Hepatocellular Carcinoma

**DOI:** 10.3389/ti.2023.11648

**Published:** 2023-09-14

**Authors:** Marco Petrus Adrianus Wilhelmus Claasen, Dimitri Sneiders, Yannick Sebastiaan Rakké, René Adam, Sherrie Bhoori, Umberto Cillo, Constantino Fondevila, Maria Reig, Gonzalo Sapisochin, Parissa Tabrizian, Christian Toso

**Affiliations:** ^1^ Department of Surgery, Division of HPB & Transplant Surgery, Erasmus MC Transplant Institute, University Medical Centre Rotterdam, Rotterdam, Netherlands; ^2^ Multi-Organ Transplant Program, University Health Network (UHN), Toronto, ON, Canada; ^3^ Centre Hépato-Biliaire, APHP Hôpital Universitaire Paul Brousse, Université Paris-Saclay, Paris, France; ^4^ Hepatology, Hepato-Pancreato-Biliary Surgery and Liver Transplantation, Fondazione IRCCS Istituto Nazionale dei Tumori, Milan, Italy; ^5^ Chirurgia Generale 2, Epato-Bilio-Pancreatica e Centro Trapianto di Fegato, Azienda Ospedale Università Padova, Padova, Italy; ^6^ General and Digestive Surgery, University Hospital La Paz, Madrid, Spain; ^7^ BCLC Group, Liver Unit, Digestive Disease Institute, Hospital Clínic, IDIBAPS CIBEREHD, University of Barcelona, Barcelona, Spain; ^8^ Liver Transplant and Hepatobiliary Surgery, Recanati/Miller Transplantation Institute, Icahn School of Medicine at Mount Sinai, New York, NY, United States; ^9^ Division of Abdominal Surgery, Faculty of Medicine, University of Geneva, Geneva, Switzerland

**Keywords:** hepatocellular carcinoma, liver transplantation, downstaging, bridging, immunotherapy

## Abstract

Liver transplantation offers the best chance of cure for most patients with non-metastatic hepatocellular carcinoma (HCC). Although not all patients with HCC are eligible for liver transplantation at diagnosis, some can be downstaged using locoregional treatments such as ablation and transarterial chemoembolization. These aforementioned treatments are being applied as bridging therapies to keep patients within transplant criteria and to avoid them from dropping out of the waiting list while awaiting a liver transplant. Moreover, immunotherapy might have great potential to support downstaging and bridging therapies. To address the contemporary status of downstaging, bridging, and immunotherapy in liver transplantation for HCC, European Society of Organ Transplantation (ESOT) convened a dedicated working group comprised of experts in the treatment of HCC to review literature and to develop guidelines pertaining to this cause that were subsequently discussed and voted during the Transplant Learning Journey (TLJ) 3.0 Consensus Conference that took place in person in Prague. The findings and recommendations of the working group on Downstaging, Bridging and Immunotherapy in Liver Transplantation for Hepatocellular Carcinoma are presented in this article.

## Introduction

Liver transplantation offers the best chance of cure for most patients with non-metastatic hepatocellular carcinoma (HCC). After their introduction in 1996, the Milan Criteria (a single lesion of ≤5 cm or 2–3 lesions of ≤3 cm) became the standard for patient eligibility for transplantation [1]. In later years, several expended selection criteria were introduced. Of these, the University of California San Francisco (UCSF) criteria (a single lesion of ≤6.5 cm or 2–3 lesions ≤4.5 cm with a total diameter ≤8 cm), the Up-to-seven criteria (the sum of the size of the largest tumor [in cm] and the number of tumors should not exceed 7), and the French AFP model (a score calculated based on a combination of AFP level, tumor size, and number which should not exceed 2) have been most widely accepted [2–4]. Post-transplant survival rates for patients transplanted within these established criteria exceed 70% at 5 years and 60% at 10 years [2, 3, 5–7]. To keep patients within these criteria while awaiting transplant and to avoid them from dropping out of the waiting list, bridging therapies such as ablation and transarterial chemoembolization (TACE) are being applied. Similarly, these treatments are used to downstage patients from outside established HCC transplant criteria to within these criteria, allowing them to become eligible for liver transplantation. When successful, downstaged patients can achieve equally meaningful post-transplant survival outcomes exceeding 65% at 5 years and 50% at 10 years [5, 8–10].

Although still in development and only recently added as part of the first-line treatment of patients with advanced HCC, immunotherapy too offers great potential in furthering the treatment of HCC [11]. Evidence for immunotherapy in neoadjuvant settings is already accumulating from early phase trials in various solid tumor types and also in HCC few studies have shown promising results, reporting major pathological response (≥70% necrosis) in 20%–42% of resected patients after receipt of neoadjuvant immunotherapy [12–15].

To address the contemporary status of downstaging, bridging, and the role of immunotherapy in both these strategies in the specific context of liver transplantation for HCC, ESOT convened a consensus conference, comprised of a global panel of expert hepatologists, transplant surgeons, and oncologists to develop guidelines on key aspects of Downstaging, Bridging and Immunotherapy in Liver Transplantation for Hepatocellular Carcinoma. The consensus findings and recommendations of these ESOT Consensus guidelines are presented in this document and are intended for healthcare providers.

## Methods

The consensus development process was governed by a dedicated ESOT Guidelines Taskforce with support from its sections, and specifically for this work the European Liver and Intestine Transplant Association (ELITA), European Transplant Allied Healthcare Professionals (ETHAP), Education Committee, Young Professionals in Transplantation (YPT), Transplant International editorial board members and patient representatives. The detailed description of methodology used is reported previously [16].

Briefly, key issues related to Downstaging, Bridging and Immunotherapy in Liver Transplantation for HCC were identified by the working group and specific clinical questions were formulated according to the PICO methodology (PICO = Population, Intervention, Comparator and Outcome) [17]. All PICO questions are listed in Table 1 and further specified in the Supplementary Material. Following the definition of the PICOs, literature searches were developed (Supplementary Material). In some, support was provided by expert staff from the Centre for Evidence in Transplantation (CET) who have expertise in conducting systematic reviews. Search strategies differed based on the type of question and whether CET was involved or not and were conducted between 14 July 2022 and 31 October 2022.

**TABLE 1:** Population, Intervention, Comparator and Outcome (PICO) questions.

1. Should all eligible patients be transplanted after successful downstaging?
2. Should all patients outside transplant criteria (all comers) be considered for downstaging?
3. Should Patients with Complete Response of HCC Macrovascular Invasion be considered for Liver Transplantation?
4. Does bridging therapy improve post-transplant survival?
5. Does bridging therapy decrease waitlist dropout?
6. Does the type of response to bridging therapy have an impact on post-transplant survival?
7. What locoregional therapy results into best short-term disease-control in HCC patients without extrahepatic disease?
8. Are patients on immunotherapy prior to liver transplantation at risk for rejection?
9. What is the best way to assess response to immunotherapy?
10. What is the safety of combined treatment with locoregional therapy and immunotherapy in the setting of transplantation?

A summary of the evidence addressing each key question by the included studies was prepared in evidence Supplementary Tables S1–S10 (Supplementary Material). The workgroup proposed a recommendation for each key question, based on the quality of evidence rated using the Grading of Recommendations Assessment, Development and Evaluation (GRADE) approach, with high quality rated as A, medium quality as B, and low quality as C; very low quality of evidence was not considered. For evaluation of the quality of evidence according to GRADE the following features were considered: study design, risk of bias, inconsistency, indirectness, imprecision, number of patients, effect, importance and publication bias [18]. Strength of recommendation was rated as 1 (strong) or 2 (weak).

Complete information including the list of consensus conference workgroup domains and process regarding consensus conference participant selection, development and refinement of consensus statements, are previously reported, in beforehand of the in-person conference held in Prague, Czech Republic, 13–15 November 2022 [16].

## Results

### 1. Should all Eligible Patients Be Transplanted After Successful Downstaging?

Currently, given the scarcity of graft resources and competing indications for liver transplantation, patients beyond conventional pre-defined criteria are often not transplanted. Despite achieving successful downstaging to within accepted criteria, patients are not always offered the option of liver transplantation. The question remains whether they should.

**Recommendation 1.1:** All HCC patients achieving a successful downstaging to pre-defined transplantable criteria should be considered for liver transplantation as the benefit in terms of both recurrence-free survival and overall survival of this approach is significantly higher than any other non-transplant strategy.
**Quality of Evidence:** High.
**Strength of Recommendation:** Strong for.
**Unmet needs:** There are no specific unmet needs. Nonetheless, additional high-quality evidence could help refine, expand and/or strengthen a future recommendation on the topic.

One single reference, a 2020 randomized controlled trial by Mazzaferro et al., met the pre-defined PICO criteria and was included for review (Supplementary Table S1) [10]. This study analyzed 74 patients from nine different Italian centers and showed that after an effective and sustained downstaging of tumors originally beyond Milan criteria, liver transplantation improved tumor event-free survival and overall survival compared with non-transplantation therapies [10]. Data supporting that successfully downstaged patients should be considered for liver transplantation.

### 2. Should all Patients Outside Transplant Criteria (All Comers) Be Considered for Downstaging?

Many patients with HCC are diagnosed at an advanced stage, falling beyond accepted transplant criteria. However, if the overall tumor burden were to decrease, they could potentially reach a stage for which liver transplantation is usually indicated. Whether this should be actively pursued, treating patients with the goal of lowering their tumor burden so that liver transplantation might become possible, regardless of their initial stage, is still up for debate.

**Recommendation 2.1:** All patients beyond transplant criteria, without extra-hepatic disease or macrovascular invasion, should be considered for downstaging as long as potentially eligible for transplantation, as the original HCC state has not demonstrated to significantly hamper post-transplant survival.
**Quality of Evidence:** Low.
**Strength of Recommendation:** Strong for.
**Unmet needs:** There are no specific unmet needs. Nonetheless, additional higher quality evidence could help refine, expand and/or strengthen a future recommendation on the topic.

After reviewing 413 references, six observational studies were found to meet the PICO criteria (Supplementary Table S2) [5, 8, 19–21]. All these six studies showed no impact of the original HCC state on post-transplant survival. Although some studies showed a trend towards decreased disease-free survival in patients with advanced HCC (based on size and number) compared to those with less advanced HCC before downstaging, none reached significance [5, 8, 20]. In addition, one study based on waitlist alpha-fetoprotein (AFP) changes even suggested the opposite, utilizing the United States (US) Scientific Registry of Transplant Recipients (SRTR) and including 60 highly selected patients. In the cohort of patients demonstrating a waitlist AFP decrease below 400 ng/mL, those with high original AFP >1,000 ng/mL showed a trend towards better post-transplant survival compared to those with original AFP between 400 and 700, and between 700 and 100 ng/mL (100% vs. ∼75% vs. ∼55%, *p* = 0.072) [19]. Altogether, the identified studies support the use of downstaging in all patients with HCC beyond conventional criteria (all comers) as long as potentially eligible for transplantation, as the post-transplant survival in case of successful downstaging is not negatively influenced by the original HCC state. Of note, data suggest that a combination of morphological and biological (AFP) criteria should be used to assess the success of downstaging in all comer patients [22]. Also, enough time should be left between a successful downstaging and transplantation (e.g., >6 months) to decrease the risk of post-transplant recurrence [22].

**Note:** The higher the burden of disease (based on morphology and/or biology), the lower the likelihood to achieve successful downstaging.
**Quality of Evidence:** Moderate.

Although the original HCC state has no demonstrated impact on post-transplant survival, several studies showed that patients with advanced HCC are more likely to fail downstaging strategies, confirming the role of downstaging as a selection tool. To illustrate, two studies including 209 and 326 patients reported rates of successful downstaging to within Milan criteria at 39.1% and 38.2% for patients originally beyond UCSF criteria, and at 58% and 45.2% for patients originally between Milan and UCSF (*p* = 0.042, *p* = 0.001) [20, 23]. However, as downstaging and palliation involve similar locoregional and systemic treatments, it can generally be argued that it is to the patients’ benefit to keep them in a downstaging strategy.

### 3. Should Patients With Complete Response of HCC Macrovascular Invasion Be Considered for Liver Transplantation?

Macrovascular invasion has historically been a contraindication for liver transplantation in patients with HCC. Although difficult to treat, some patients with macrovascular invasion manage to achieve complete radiologic response after locoregional or systemic treatment. Whether these patients should be considered for liver transplantation is still to be answered.

**Recommendation 3.1:** There is insufficient evidence to recommend or not recommend liver transplantation for patients with HCC macrovascular invasion with complete response to therapy.
**Quality of Evidence:** Low.
**Strength of Recommendation:** N/A.
**Unmet needs:** Outcomes for patients with HCC and macrovascular invasion transplanted after complete response by pre-operative therapy are missing. Therefore, future studies should focus on neoadjuvant locoregional or systemic therapies and sustained (∼6 months) complete response. In this effort, differences in type of portal vein tumor thrombus (Vp1-Vp4) should also be compared.

Of the 85 references found, seven studies met all pre-defined PICO criteria. After reviewing their references, one more study was identified for inclusion, bringing the total to eight studies for further review (Supplementary Table S3) [24–30]. Although several studies demonstrated a 5 years overall survival rate of more than 50% in patients who received downstaging treatments before transplantation, most studies also reported high recurrence rates [24–30]. The largest included study, by Yu et al., analyzed 176 patients with portal vein tumor thrombus (PVTT) type 1–2 and showed a 5 years overall survival of 78.3% in patients with type 1 PVTT compared to 51.6% for those with type 2 PVTT (*p* = 0.005) [28]. However, recurrence-free survival was about 46% in both groups. Moreover, no subgroup analysis was performed for patients who achieved complete response after pre-operative therapy. This subgroup analysis was also lacking in most of the other included studies [24, 25, 29, 30]. The two studies that did report on outcomes for patients with radiologic (near-to) complete response, by Soin et al. (*n* = 25) and Serenari et al. (*n* = 5), showed a 5 years overall survival of 57% and 60%, and a recurrence rate of 24% and 60%, respectively [26, 27]. Consequently, due to insufficient evidence in the contemporary literature, no clear recommendation can be made on whether or not patients with HCC and macrovascular invasion should be considered for transplantation after complete radiologic response. If pursued, this strategy should be carried out within specific clinical trial settings.

### 4. Does Bridging Therapy Improve Post-Transplant Survival?

Bridging therapy is commonly used to keep patients with HCC within established transplant criteria. However, it is uncertain whether this also results in improved post-transplant survival and should therefore be standard practice for every patient on the transplant waiting list.

**Recommendation 4.1:** There are some studies that suggest a positive effect of bridging therapy on long-term post-transplant survival. Therefore, bridging therapy should be considered in patients if feasible.
**Quality of Evidence:** Low.
**Strength of Recommendation:** Strong for.
**Unmet needs:** There are no specific unmet needs. Nonetheless, additional higher quality evidence could help refine, expand and/or strengthen a future recommendation on the topic.

After screening 989 references, eight studies were selected for full review. One was a systematic review and meta-analysis (the studies analyzed herein were not separately reinstated for full review), the remaining seven were observational studies (Supplementary Table S4) [31–38]. Some of the identified studies showed significantly better long-term post-transplant survival outcomes in patients treated with bridging therapy [33, 35, 37, 38]. The largest of these studies, by Xing and Kim, looked at 14.511 transplanted patients within Milan criteria pre-transplant (3.889 with bridging, 10.622 without) and showed a 1, 3, and 5 years post-transplant survival of 95%, 85%, 80% in bridged patients versus 94%, 83%, 78% in patients without bridging (*p* < 0.001) [37]. In the multivariable analysis, bridging therapy remained associated with a significantly better post-transplant survival with a hazard ratio (HR) of 2.28 (95% CI 1.39–3.14; *p* = 0.003). Bauschke et al. showed in their cohort of 70 patients, all within Milan criteria, that the survival benefit persists even after 10 years post-transplant (95% bridged vs. 73% without bridging, *p* = 0.014) [33]. Another study analysing patients classified as within Milan criteria pre-transplant showed that the positive effect of bridging therapy on post-transplant survival even seems to last in a setting of recurrence, where the median survival of recurred bridged patients was 75.9 months versus 53.1 months in patients without bridging treatment (*p* = 0.001) [35]. Looking specifically at patients within UCSF criteria, two studies were evaluated, one with 134 patients and another with 39 patients, but both failed to report any statistical difference in survival between bridged and non-bridged patients.

### 5. Does Bridging Therapy Decrease Waitlist Dropout?

It is widely believed that bridging therapy is effective in keeping patients within established transplant criteria, however, whether it actually results in reduced waitlist dropout has yet to be confirmed.

**Recommendation 5.1:** Due to inherent confounding in the indication to bridge, evidence in the current literature is insufficient to identify whether or not bridging therapy decreases waitlist dropout. Therefore, no recommendation can be made.
**Quality of Evidence:** Low.
**Strength of Recommendation:** N/A.
**Unmet needs:** To determine whether bridging therapy actually results in a reduction in waitlist dropout, avoiding the currently inherited confounding in the indication to bridge, a randomized controlled trial would be required. However, with the current assumption that bridging therapy, already standard practice, is effective in keeping patients within transplant criteria, such a trial is considered ethically unjustifiable.

A total of 634 references were identified, of which six observational studies and one systematic review and meta-analysis met the pre-defined PICO criteria (the studies analyzed in the systematic review were not separately reinstated for review) (Supplementary Table S5) [31, 34, 36, 38–41]. Considering the most common transplant criteria (Milan, UCSF, ETC), none of the identified studies showed a decrease in overall or disease-specific waitlist dropout for patients who received bridging treatment compared to those without bridging treatment [31, 34, 36, 38–41]. Although not statistically significant, some of the studies did show a longer waitlist time in the group of patients who received bridging therapy [34, 36, 39, 40]. When specifically focussing on progression-related waitlist dropout, one study—evaluating 265 patients within Milan criteria—showed a statistically significantly lower dropout rate in the bridged patient population (2.58%) versus patients without bridging therapy (8.18%) [38]. However, the all-cause waitlist dropout in this study was higher in the bridged patient group (28.4% vs. 14.5% without bridging). Another study, a 2018 meta-analysis by Kulik and others, evaluating 257 cirrhotic patients classified as T2 HCC (patients within Milan criteria), reported no difference in progression-related waitlist dropout between groups treated with and without bridging treatment (relative risk [RR] 0.32; 95% confidence interval 0.06–1.85) [31]. Whether the type of bridging therapy plays a role in waitlist dropout was evaluated in the study by Györi et al., where they analyzed 84 patients within Milan criteria [34]. A transarterial chemoembolization (TACE)-based group (*n* = 48) was compared with a percutaneous ethanol injection (PEI)/radiofrequency ablation (RFA) group (*n* = 32) and a control group consisting of patients without bridging treatment (*n* = 22). They found no difference in all-cause waitlist dropout between groups: 41.7% TACE-based vs. 31.2% PEI/RFA vs. 36.4% control (*p* = 0.65) [34]. However, a serious limitation in all these retrospective studies, is the inextricable involvement of selection bias in the indication for bridging. Consequently, bridged and non-bridged populations consistently include non-comparable groups of patients and therefore ineluctably mask any effect that bridging therapy might have on waiting list dropout. Thus, precluding the effect of bridging on waitlist dropout from being inferred.

### 6. Does the Type of Response to Bridging Therapy Have an Impact on Post-Transplant Survival?

Bridging therapies are used in several patients within conventional transplant criteria to delay tumor progression and to minimize the risk of de-listing while on the waiting-list (dropout). Despite the strong belief that the type of response to bridging is able of influencing the rate of post-transplant tumor recurrence, this, and the weight that tumor response may have on post-transplant survival, have yet to be determined.

**Recommendation 6.1:** The aim of all bridging treatments carried out on the waiting-list should be to achieve a complete pathological response as this has shown to be associated with both improved recurrence-free and overall survival. Since there is no radiological imaging yet able of accurately predicting post-transplant complete pathologic response, sustained radiologic response may be considered as the best surrogate to pursue in the pre-transplant setting.
**Quality of Evidence:** Low.
**Strength of Recommendation:** Strong for.
**Unmet needs:** There are no specific unmet needs. Nonetheless, additional higher quality evidence could help refine, expand and/or strengthen a future recommendation on the topic.

Given the high rate of overestimation of treatment response of radiology over pathology, the literature review focused on pathologic responses only. After the identification of 423 references, nine references were included for further review (Supplementary Table S6) [35, 42–49]. All but one study analyzed outcomes achieved after both bridging and downstaging therapies, with TACE being the most commonly used treatment modality. In all studies, patients with complete pathologic response at explant pathology showed better overall survival and recurrence-free survival rates compared with those without complete pathological response [35, 42–49]. Allard et al. found that the favorable prognostic effect of response induced by TACE on explant pathology in 189 patients was confirmed not just for complete necrosis but also for “near to complete responses” (>90%), suggesting a “nearly all - or nothing” rule [48]. This data was later confirmed by the largest single-center US experience (*n* = 501) published by Agopian in the same year, updated in 2020 in a multicentric fashion including 3,439 patients undergoing liver transplantation from 2002 to 2013 in 20 US centers and all receiving bridging and/or downstaging therapies pre-transplant (with 802 patients showing complete pathological response) [43, 49]. All data supporting the need to pursue a complete (or close to complete) radiological tumor response in patients with HCC listed for liver transplantation.

### 7. What Locoregional Therapy Results Into Best Short-Term Disease-Control in HCC Patients Without Extrahepatic Disease?

Many different types of locoregional therapy for HCC exist. In the context of liver transplantation, locoregional therapy is used in the attempt to effectively control the patient’s tumor burden until a suitable liver donor becomes available for transplantation. Consequently, adequate short-term disease control is desired. What type of locoregional therapy best achieves this remains to be determined.

**Recommendation 7.1:** Specifically for waitlisted patients, no recommendation can be made due to the absence of unconfounded evidence. Therefore, the type of locoregional therapy should be selected according to patient and center characteristics using multidisciplinary assessment. Although data outside a transplant setting cannot be translated directly to waitlisted patients, they can provide guidance in determining which treatment might be advisable for different patients (Table 2).
**Quality of Evidence:** Low.
**Strength of Recommendation:** N/A.
**Unmet needs:** To determine what locoregional therapy results into best short-term disease-control in waitlisted HCC patients, avoiding both selection bias and the many patient-related confounders, randomized controlled trials would be required. However, given many patient-related and treatment-related confounders determine whether certain types of locoregional therapies can be applied to selected patients with HCC, accruing enough patients in such trials will be extremely difficult.

**TABLE 2:** Guidance document for determining the best locoregional treatment approach for short-term disease control in patients with HCC based on randomized controlled trials of locoregional treatment in a non-transplant setting.

Lesion number	Lesion size	Supporting statements
1	3–5 cm	1. When feasible, liver resection, preferably by laparoscopic route and segmental extension, should be considered
		Level of evidence: Moderate
		Level of recommendation: Weak for
		2. When technically feasible RFA or MWA are the preferred second line therapies and are equally effective in obtaining short-term tumor control. When ablation is not obtained or not expected to be obtained, TACE is the preferred therapy
		Level of evidence: Moderate
		Level of recommendation: Weak for
		3. Intention to treat with combined RFA/MWA and TACE may result in superior short term tumor control compared to TACE or RFA alone and can be used on indication
		Level of evidence: Low
		Level of recommendation: Weak for
		4. Alternatives to TACE or RFA/MWA, including radio-embolization or SIRT, SBRT, proton-beam radiation therapy or brachytherapy have shown non-inferior or improved short term tumor control in preliminary trials and should preferably be used in a research setting
		Level of evidence: Low
		Level of recommendation: Weak for
≤3	≤3 cm	1. RFA or MWA is the preferred first line therapy and are equally effective in obtaining short-term tumor control
		Level of evidence: Moderate
		Level of recommendation: Strong for
		2. Intention-to-treat with combined ablation therapy and TACE does not impact short term tumor control
		Level of evidence: Low
		Level of recommendation: Weak for
≥1	≥5	1. Liver resection, if feasible and indicated, is associated to the higher probability to obtain a complete response on the single HCC
		Level of evidence: Low
		Level of recommendation: Weak for
		2. Downstaging therapy with TACE is preferred over bland embolization or chemo infusion alone
		Level of evidence: Low
		Level of recommendation: Weak for
		3. Intention to treat with combined RFA/MWA and TACE may result in superior short term tumor control than TACE alone and can be used on indication.
		Level of evidence: Low
		Level of recommendation: Weak for
		4. Alternatives to TACE, including radio-embolization or SIRT, SBRT, proton-beam radiation therapy or brachytherapy have shown non-inferior or slightly improved short term tumor control in preliminary trials and should preferably be used in a research setting
		Level of evidence: Low
		Level of recommendation: Weak for

As treatment allocation in clinical practice is subjected to both confounding factors and selection bias, only randomized controlled trials (RCTs) on the application of locoregional therapies outside a transplant setting were included. This approach allows for the least biased comparison between therapeutic modalities. Of the 2,944 unique references found, 40 RCTs comparing at least two treatment modalities were included for further review (Supplementary Table S7) [50–88]. Treatment comparisons were grouped according to lesion size and number combinations.

RCTs on uninodular lesions with size up to 3 cm (BLCL 0, A; within Milan) have compared: radiofrequency ablation (RFA) to percutaneous ethanol injection (PEI) [52, 57, 68, 74, 77], RFA to percutaneous laser ablation (PLA) [80], RFA to percutaneous acetic acid injection (PAAI) [57, 64], RFA to cryoablation [55], RFA to microwave ablation (MWA) [50, 58, 59, 69, 73, 76], and RFA to RFA combinatorial approaches [61, 65, 70, 71, 78, 82]. RFA appeared to induce higher frequencies of radiological complete responses (rCR) and improved 1 year local recurrence (LR) rate compared to PEI and PLA. Compared to PAAI, RFA induced similar rCR. However, 3 years LR rate was improved in RFA-treated versus PAAI-treated patients (RR = 0.41, 95% CI: 023–0.91) [57]. Cryoablation has been shown to have equal rCR, 1 year LR rate, 1-year overall survival, and 1 year disease-free survival as RFA, albeit in a single RCT [55]. In a meta-analysis on RCTs among RFA- and MWA-treated lesions no difference in radiological complete response rates was observed (RR: 1.01, 95% CI: 0.99–1.02) [67]. Moreover, 1 year disease-free and overall survival rates were similar. No difference in adverse events (Aes) could be observed between RFA and MWA-treated patients. RCTs on combination of RFA with TACE [65, 70, 82] or other therapeutic regimen (PEI [71], Iodine-125 [78], Interferon alpha [61]) did not show or report any difference in rCR in these tumor lesions compared to RFA only.

RCTs comparing RFA to PEI [52, 74], RFA to PLA [80], RFA to PAAI [64], RFA to cryoablation [55], and RFA to MWA [50, 58, 59, 69, 73, 76] have included uninodular lesions, ranging 3–5 cm as well. As RFA and MWA in these trials have shown to be clinically effective one might suggest that these techniques are preferred as first line regimen. Yet, locoregional ablative therapies tend to become less effective if tumor lesion size increases.

In case of increased tumor burden, intra-arterial therapies or radiotherapy provide an alternative. Different RCTs on uninodular lesions ranging 3–5 cm (BCLC A; within Milan) and uni-/multinodular lesions ≥5 cm (BCLC A, outside Milan; BCLC B, outside Milan, resp.) have compared: TACE to transarterial or “bland” embolization (TAE) [54, 60, 81, 84], TACE/RFA to TACE combined with RFA [51, 56, 65, 66, 70, 72, 86], TACE to transarterial radio-embolization (TARE) [53, 62, 83, 88], TACE to transarterial ethanol ablation (TAEA) [75], TACE to transarterial chemo-infusion (TACI) [63], and TACE to radiotherapy [79, 85]. Hyperselective TACE (tend to) induced higher frequencies of rCR or radiological partial response (rPR) compared to “bland” embolization. 1 year disease-free and overall survival was either non-significantly different among the groups or tended to be increased in TACE-treated patients. When combining TACE with ablative therapies, combination regimen appeared to induce higher rCR (i.e., + PEI [66], + RFA [86], and + cryoablation [51]), 1 year disease-free survival [66], and 1-year overall survival [51, 86], although studied in relatively small cohorts. RCTs comparing TACE to TARE have shown conflicting results. Whereas Raoul et al. reported no difference in rCR/PR when using Iodine-131 radioembolisation [53], other trials have shown a trend to higher radiological response rates in Yttrium-90 (Y-90) radioembolization cohorts compared to TACE [83, 88]. Moreover, Salem et al. have observed that Y-90 appeared to have lower 1 year LR rate [62]. Generally, treatment-related or grade ≥3 AEs were either equal or reduced in favor of TARE. Conformably, in the prospective, multi-center, non-randomized MERITS-LT trial both TACE and Y90-TARE showed equal efficacy in downstaging towards liver transplantation [87]. Though not statistically significant, explanted livers of TARE-treated patients demonstrated higher frequencies of tumor necrosis (30.8% vs. 20.5%) and lower frequencies microvascular invasion (7.7% vs. 20.5%) hinting towards improved local tumor control. Nowadays, TARE has been accepted as an effective alternative in case TACE is contraindicated (e.g., portal thrombosis). To this end, no clear benefit of TAEA, TACI, or radiotherapy (i.e., proton-beam, brachytherapy) over TACE in RCTs was observed. Yet, recent prospective cohort studies strongly hint to safe and superior efficacy of stereotactic body radiotherapy over TACE as bridge to transplant [89, 90]. Any conclusive results on these therapies are expected from ongoing phase III RCTs (i.e., NCT03960008).

Although this data provides valuable insight in the potential of each locoregional treatment in a non-transplant setting, their results cannot directly be translated to waitlisted patients. Therefore, no recommendations can be made. Nonetheless, these comparisons can provide guidance in determining the kind of treatment to pursue (Table 2).

### 8. Are Patients on Immunotherapy Prior to Liver Transplantation at Risk for Rejection?

Immunotherapy has recently become part of the standard treatment for advanced unresectable HCC who are not amenable to curative or locoregional therapy. Due to its promising results, interest has emerged in the use of immunotherapy in a neoadjuvant setting. Whether patients receiving immunotherapy prior to liver transplantation are at risk for rejection has yet to be determined.

**Recommendation 8.1:** Due to insufficient evidence, no meaningful recommendation can be made.
**Quality of Evidence:** Low.
**Strength of Recommendation:** N/A.
**Unmet needs:** (1) Further investigations are needed to explore the safety and long-term oncologic outcomes in the pre-transplant setting. (2) Patient selection for immune checkpoint inhibitors (ICI), minimal washout period between the last drug dose and transplantation, observation period, biomarkers are unmet clinical needs that require investigation.

Of the 1,560 references identified, nine studies on liver transplantation in patients previously treated with immune checkpoint inhibitors were included, representing 27 cases (Supplementary Table S8) [91–99]. The first case reported resulted in fatal hepatic necrosis at day 8th and patient loss [91]. The ICI was given within 4 weeks before transplantation. A minimum washout period (4 weeks) prior to transplantation given the half-life of 27 days was proposed. Subsequent reports have shown successful results [92, 94–99]. In total, four cases of severe rejection were reported with two successful re-transplantations [91, 93, 97, 98]. Since drug type, pre-transplant treatment and dosage, tumor burden, and response vary from case to case, further investigations are needed to explore the safety and long term oncologic outcomes in a pre-transplant setting.

### 9. What is the Best Way to Assess Response to Immunotherapy?

To optimize the use of immunotherapy treatment in patients with HCC and to be able to evaluate its effect in a (neo)adjuvant setting, it is imperative that tumor response after immunotherapy can be adequately assessed. However, the best way to do this has yet to be determined.

**Recommendation 9.1:** There is insufficient evidence to make any meaningful recommendation on how best to assess response to immunotherapy for HCC.
**Quality of Evidence:** Low.
**Strength of Recommendation:** N/A.
**Unmet needs:** (1) Improved imaging techniques and biomarkers are needed to define response ahead of pathologic assessment and oncologic outcomes. (2) Explant analysis of specimens should be done prospectively with careful radiology-pathology correlation.

After an extensive review of 6,800 references, seven studies were selected for inclusion (Supplementary Table S9) [11, 13–15, 100–102]. Radiologic evaluation of response after immunotherapy is primarily derived from the recent trials on immunotherapy within advanced HCC where survival benefit was associated with objective response and significant reduction in tumor burden [11, 100–102]. In these studies, the objective response rate by mRECIST ranged from 22% to 34%, whereas complete response was reported in 2.2%–5.5% of the cases [11, 100–102]. Unfortunately, these studies lack confirmation of actual response through pathological assessment. Three recent trials that published on the use of neoadjuvant therapy prior to resection in HCC did report on both response seen on imaging and determined by pathologic assessment. Complete pathologic response ranged from 8% to 25% and major pathologic response (>70% necrosis) was seen in 20%–42%, while pre-operative imaging according to RECIST 1.1 reported partial and complete response in only 8%–15% and 0%, respectively [13–15]. Although data on imaging-pathology response correlations in a transplantation setting are lacking, encouraging pathologic response rates have been reported. In a study of 9 patients who underwent ICI in combination with locoregional therapy, downstaging was successful in 4/5 patients and major pathologic response (>70% necrosis) was noted in 6/9 patients [96]. Improved imaging techniques and biomarkers are needed to define response ahead of pathologic assessment and oncologic outcomes. Given the high rate of explants exceeding Milan criteria post transplantation, significant limitations occur with the current contrast enhanced computed tomography (CT) and magnetic resonance imaging techniques (MRI) in predicting treatment response [87]. In addition, with the use of immunotherapies, the immunologic changes within the tumor and tumor microenvironment may impact the relation between the degree of pathologic and radiographic response [14]. Moreover, the vasoconstrictive and antiangiogenic effects of the drugs may induce a false positive assessment of response by mRECIST [103, 104].

### 10. What Is the Safety of Combined Treatment With Locoregional Therapy and Immunotherapy in the Setting of Transplantation?

A combined treatment of immunotherapy and locoregional therapy may be more effective than each treatment separately. However, it remains to be seen whether such combined treatment approach is safe in the context of transplantation.

**Recommendation 10.1:** Since there is no data in the context of pre- or post-liver transplantation, no recommendation can be made.
**Quality of Evidence:** N/A.
**Strength of Recommendation:** N/A.
**Unmet needs:** Further investigations that explore the safety and long-term oncologic outcomes in the pre- and post-transplant setting are needed.

Since no data was found on combined treatment with locoregional and immunotherapy in the setting of transplantation, data outside transplant setting was assessed. In this context, a total of 450 references were identified, whereas 14 were eventually included for further review (Supplementary Table S10) [105–119]. Two of these were systematic reviews and meta-analyses [105, 106]. The first, including 19 studies and comparing TACE or RFA with immunotherapy, did not evaluate safety profiles [105]. The second, including four studies comparing TACE with dendritic cells therapy, reported that patients in the TACE-DC-CIK group were more likely to suffer a fever than the ones in the control group (*p* = 0.001). In the five prospective studies, one randomized controlled trial and four non-randomized trials, no safety difference between arms was reported [110–115]. However, the small sample sizes limited the robustness of their conclusion. Finally, of the seven non-randomized retrospective studies, five focused on early-death or severe complications with none of the studies reporting any major complication or death associated with the treatment evaluated [108, 117–120]. In the remaining two retrospective studies safety was not reported [107, 109]. Although these data provide valuable insight into the safety and long-term oncologic outcomes of combined treatments of locoregional therapy and immunotherapy in a non-transplant setting, they cannot be extrapolated to a transplant/waitlist-setting. Therefore, no recommendation can be made.

## Summary and Future Considerations

The Transplant Learning Journey (TLJ) 3.0 consensus conference resulted in several recommendations pertaining to Downstaging, Bridging and Immunotherapy in Liver Transplantation for HCC. Starting with downstaging. Though not always successful, downstaging should always be aimed for regardless of disease burden as the original HCC state has demonstrated little impact on post-transplant survival. Moreover, as downstaging and palliation involve similar locoregional and systemic treatments, it can generally be argued that it is to the patients’ benefit to keep them in a downstaging strategy. If successful downstaging has been achieved, patients should always be considered for liver transplantation as the benefit in terms of both recurrence-free and overall survival of this approach is significantly higher than any other non-transplant strategy. Although liver transplantation for patients with macrovascular invasion has been shown to be feasible, recurrence rates are generally high, necessitating further investigation to determine whether patients with HCC and macrovascular invasion should be considered for liver transplantation if complete radiologic response has been achieved. In the context of bridging, some studies suggest a positive effect of bridging therapy on long-term post-transplant survival and therefore should be considered if feasible. When applied, the aim should be to attain complete response, as a complete pathological response has shown to be associated with improved recurrence-free and overall survival. Since radiological imaging is not able to accurately predict post-transplant complete pathologic response, sustained radiologic response may be considered as the best surrogate to pursue in the pre-transplant setting. Unfortunately, whether or not bridging therapy decreases waitlist dropout cannot be determined from the contemporary literature due to inherent confounding in the indication to bridge. In terms of the type of bridging therapy to use, selection should be made according to patient and center characteristics using multidisciplinary assessment. Finally, although immunotherapy has shown promising results, further investigations are needed to explore its safety (rejection) and long-term oncologic outcomes in a pre-transplant setting, as well as which patients to select, the minimal washout period between the last drug dose and transplantation, and the optimal duration of observance. The same holds for immunotherapy use in a pre- or post-transplant setting when combined with locoregional treatments. To support research in these areas, improved imaging techniques and biomarkers are needed to define immunotherapeutic response ahead of pathologic assessment and oncologic outcomes.

## Data Availability

The original contributions presented in the study are included in the article/Supplementary Material, further inquiries can be directed to the corresponding author.

## Collaborators

Helena Degroote (Department of Hepatology and Gastroenterology, Ghent University Hospital, Belgium); Andrea Della Penna, Silvio Nadalin (Department of General, Visceral and Transplant Surgery, University Hospital Tübingen, Tübingen, Germany); Emmanouil Giorgakis (Division of Transplantation, Department of Surgery, University of Arkansas for Medical Sciences, Little Rock, AR, United States); Oleg Godik (Department of Pediatric Surgery & Urology, National Medical University and Clinic Oberig, Kiev, Ukraine); Mihnea-ioan Ionescu (The Liver Unit, Queen Elizabeth Hospital Birmingham, University Hospitals Birmingham NHS Foundation Trust, Birmingham, United Kingdom); Giulia Magini (Division of Abdominal Surgery, Faculty of Medicine, University of Geneva, Geneva, Switzerland); Wojtek Polak (Department of Surgery, Division of HPB & Transplant Surgery, Erasmus MC Transplant Institute, University Medical Centre Rotterdam, Rotterdam, Netherlands); Karen Rockell (Co-Director and PPIE Lead of the UK Organ Donation and Transplantation Research Network, London, United Kingdom); Gianluca Rompianesi (Department of Clinical Medicine and Surgery, Federico II University Hospital, Naples, Italy); Ian Rowe (Leeds Institute for Medical Research, University of Leeds, Leeds, United Kingdom); Stratigoula Sakellariou (First Department of Pathology, National and Kapodistrian University of Athens, Laiko General Hospital, Athens, Greece); Katharina Staufer (Department of Internal Medicine III, Division of Gastroenterology & Hepatology, Medical University of Vienna, Austria; Department of General Surgery, Division of Transplantation, Medical University of Vienna, Austria); Luca Toti (HPB and Transplant Unit, Department of Surgery, University of Rome Tor Vergata, Rome, Italy).

## Glossary

AE: adverse events
AFP: alpha-fetoprotein
CET: Centre for Evidence in Transplantation
CT: computed tomography
DFS: disease-free survival
ELITA: European Liver and Intestine Transplant Association
ESOT: European Society of Organ Transplantation
ETHAP: European Transplant Allied Healthcare Professionals
GRADE: Grading of Recommendations Assessment, Development and Evaluation
HCC: hepatocellular carcinoma
HR: hazard ratio
LR: local recurrence
LT: liver transplantation
MRI: magnetic resonance imaging
MWA: microwave ablation
N/A: not applicable
OS: overall survival
PAAI: percutaneous acetic acid injection
PEI: percutaneous ethanol injection
PICO: Population, Intervention, Comparator and Outcome
PLA: percutaneous laser ablation
ICI: immune checkpoint inhibitors
PVTT: portal vein tumor thrombus
rCR: radiological complete response
RCT: randomized controlled trial
RFA: radiofrequency ablation
RFS: recurrence-free survival
rPR: radiological partial response
RR: relative risk
SRTR: Scientific Registry of Transplant Recipients
TACE: transarterial chemoembolization
TACI: transarterial chemo-infusion
TAE: transarterial embolization
TAEA: transarterial ethanol ablation
TARE: transarterial radio-embolization
TLJ: Transplant Learning Journey
UCSF: University of California San Francisco
US: United States
Y-90: Yttrium-90
YPT: Young Professionals in Transplantation
