# Supplementary material for: European Society of Organ Transplantation (ESOT) Consensus Report on Downstaging, Bridging and Immunotherapy in Liver Transplantation for Hepatocellular Carcinoma
Source: Transpl Int. 2023 Sep 14;36:11648. doi: 10.3389/ti.2023.11648 (PMC10533675; doi:10.3389/ti.2023.11648)
Supplement: Supplementary file 1 [file DataSheet2.pdf]

Supplementary material

Capsule Sentence Summary

Based on a review of the contemporary literature, recommendations on downstaging, bridging, and immunotherapy in liver transplantation for HCC were formulated, discussed, and put to a vote at the ESOT TLJ Consensus Conference. The final recommendations are presented in this article.

PICO questions

<u>PICO 1: Should all eligible patients be transplanted after successful downstaging?</u>

Population: HCC patients with successful downstaging (=reaching transplant criteria)

Intervention: Listing in view of transplantation

Comparators: No transplantation

Outcome: Intend-to-treat survival

Paper type: RCT

<u>PICO 2: Should all patients outside transplant criteria (all comers) be considered for downstaging?</u>

Population: HCC patients with successful downstaging (=reaching transplant criteria)

Intervention: Patients originally “just” outside transplant criteria

Comparators: Patients originally “far” outside transplant criteria

Outcome: Intend-to-treat survival

<u>PICO 3: Should patients with complete response of HCC macrovascular invasion be considered for liver transplantation?</u>

Population: Liver transplant recipients with complete response of HCC macrovascular invasion

Intervention: Listing in view of transplantation

Comparators: No transplantation

Outcome: Intend-to-treat survival

<u>PICO 4: Does bridging therapy improve post-transplant survival?</u>

Population: Liver transplant candidates with HCC

Intervention: Bridging (all types)

Comparator: No bridging

Outcome: Post-transplant overall survival

PICO 5: Does bridging therapy decrease waitlist dropout?

Population: Liver transplant candidates with HCC

Intervention: Bridging (all types)

Comparator: No bridging

Outcome: Waitlist drop-out (including both list exclusion/mortality)

PICO 6: Does the type of response to bridging therapy have an impact on post-transplant survival?

Population: Liver transplant candidates with HCC

Intervention: Complete radiological or pathological response

Comparators: No response (excluding progression)

Outcome: Post-transplant survival

PICO 7: What locoregional therapy results into best short-term disease-control in HCC patients without extrahepatic disease?

Population: Patients with HCC

Intervention: Ablation

Comparators: TACE, SBRT, resection, SIRT

Outcome: Clinical response rate

Paper type: RCT

PICO 8: Are patients on immunotherapy prior to liver transplantation at risk for rejection?

Population: Patients with HCC treated by immunotherapy prior to transplantation

Comparators: Patients with HCC not treated by immunotherapy prior to transplantation

Outcome: Rejection rate

PICO 9: What is the best way to assess response to immunotherapy?

Population: Patients with HCC treated with immunotherapy

Gold-standard: CT

Comparators: MRI

Outcome: Predictor of pathological response (% of necrotic tumor area)

Secondary outcome: Progression-free survival

PICO 10: What is the safety of combined treatment with locoregional therapy and immunotherapy in the setting of transplantation?

Population: Patients with HCC

Intervention: Combined immunotherapy and LRT

Comparators: LRT

Outcome: Side effects (grade 3 and above)

Secondary outcome: Intend-to-treat survival

Literature searches

PICO 1: Should all eligible patients be transplanted after successful downstaging?

A review of the MEDLINE database was carried out until September 30, 2022. The search strategy used included:

1. Carcinoma, Hepatocellular (HCC)
2. Liver Transplantation
3. Downstaging

PICO 2: Should all patients outside transplant criteria (all comers) be considered for downstaging?

A review of the MEDLINE and EMBASE databases was carried out until July 31, 2022. The search strategy used included:

1. Carcinoma, Hepatocellular
2. Liver Transplantation
3. Downstaging

PICO 3: Should patients with complete response of HCC macrovascular invasion be considered for liver transplantation?

A review of PubMed was carried out until July 14, 2022. The search query used was: (regression[tiab] OR response[tiab] OR downstaging[tiab] OR downstaged[tiab] OR cured[tiab] OR cure[tiab]) AND ("Carcinoma, Hepatocellular"[Mesh] OR HCC[tiab] OR (hepatocellular[tiab] AND (carcinoma[tiab] OR cancer[tiab]))) AND ("Liver Transplantation"[Mesh] OR (liver[tiab] AND (transplant[tiab] OR transplantation[tiab]))) AND ("Vascular Neoplasms"[Mesh] OR

"Thrombosis"[Mesh] OR macrovascular invasion[tiab] OR macroscopic vascular invasion[tiab] OR (portal vein[tiab] AND (tumor[tiab] OR tumour[tiab]) AND (thrombus[tiab] OR thrombosis[tiab]))) AND 2012/01/01:2022/07/14[dp]

PICO 4: Does bridging therapy improve post-transplant survival?

The Transplant Library (TL), MEDLINE and EMBASE were searched from 1996 to July 14, 2022. The TL includes all randomized controlled trials and systematic reviews in the field of solid organ transplantation published as full text or in abstract form, sourced mainly from MEDLINE/PubMed and hand-searches of congress proceedings. The search strategy used was:

1. Carcinoma, Hepatocellular/su, th, tr [Surgery, Therapy, Transplantation]
2. (hepatocellular cancer$ or hepatocellular carcinoma$ or hepatic cancer$ or HCC).ti,ab.
3. or/1-2
4. Liver Transplantation/
5. liver transplant$.ti,ab.
6. or/4-5
7. Chemoembolization, Therapeutic/
8. Ablation Techniques/
9. (bridg$ or ablation or RFA or MWA or RFT or transarterial chemo?embolization or transarterial chemo?embolisation or TACE or TACI or TAE or radio?embolization or radio?embolisation or TARE or intra?arterial microbrachytherapy or intraarterial microbrachytherapy or selective internal radiation therapy or SIRT or resect$ or stereotactic body radio?therapy or SBRT or percutaneous ethanol injection or PEI).ti,ab.
10. or/7-9
11. bridg$.mp.
12. 3 and 6 and 10 and 11
13. (survival or mortality or death or die$).mp.
14. 12 and 13
15. limit 14 to yr="1996 - Current"
16. limit 15 to english language
17. animals/
18. 16 not 17

PICO 5: Does bridging therapy decrease waitlist dropout?

The Transplant Library (TL), MEDLINE and EMBASE were searched from 1996 to July 14, 2022. The TL includes all randomized controlled trials and systematic reviews in the field of solid organ transplantation published as full text or in abstract form, sourced mainly from MEDLINE/PubMed and hand-searches of congress proceedings. The search strategy used was:

1. Carcinoma, Hepatocellular/su, th, tr [Surgery, Therapy, Transplantation]
2. (hepatocellular cancer$ or hepatocellular carcinoma$ or hepatic cancer$ or HCC).ti,ab.
3. or/1-2

4. Liver Transplantation/
5. liver transplant$.ti,ab.
6. or/4-5
7. Chemoembolization, Therapeutic/
8. Ablation Techniques/
9. (bridg$ or ablation or RFA or MWA or RFT or transarterial chemo?embolization or transarterial chemo?embolisation or TACE or TACI or TAE or radio?embolization or radio?embolisation or TARE or intra?arterial microbrachytherapy or intraarterial microbrachytherapy or selective internal radiation therapy or SIRT or resect$ or stereotactic body radio?therapy or SBRT or percutaneous ethanol injection or PEI).ti,ab.
10. or/7-9
11. bridg$.mp.
12. 3 and 6 and 10 and 11
13. (mortality or death or die$ or drop?out or withdr?w$ or remov$ or delist$).mp.
14. 12 and 13
15. limit 14 to yr="1996 - Current"
16. limit 15 to english language
17. animals/
18. 16 not 17

<u>PICO 6: Does the type of response to bridging therapy have an impact on post-transplant survival?</u>

A review of the MEDLINE database was carried out until July 31, 2022. The search strategy used included:

1. Carcinoma, Hepatocellular (HCC)
2. Liver Transplantation
3. Bridging
4. Necrosis

<u>PICO 7: What locoregional therapy results into best short-term disease-control in HCC patients without extrahepatic disease?</u>

A review of MEDLINE, EMBASE, Cochrane, Web of Science, and Google Scholar was carried out until August 31, 2022. The search strategy used was:

1. Carcinoma, Hepatocellular/su, th, tr [Surgery, Therapy, Transplantation]
2. (hepatocellular cancer$ or hepatocellular carcinoma$ or hepatic cancer$ or HCC).ti,ab.
3. or/1-2
4. Liver Transplantation/
5. liver transplant$.ti,ab.
6. or/4-5
7. Chemoembolization, Therapeutic/
8. Ablation Techniques/
9. (bridg$ or ablation or RFA or MWA or RFT or transarterial chemo?embolization or transarterial chemo?embolisation or TACE or TACI or TAE or radio?embolization or

radio?embolisation or TARE or intra?arterial microbrachytherapy or intraarterial microbrachytherapy or selective internal radiation therapy or SIRT or resect$ or stereotactic body radio?therapy or SBRT or percutaneous ethanol injection or PEI).ti,ab.
10. or/7-9
11. bridg$.mp.
12. 3 and 6 and 10 and 11
13. (survival or mortality or death or die$).mp.
14. respon$.mp.
15. 12 and 13 and 14
16. limit 15 to yr="1996 - Current"
17. limit 16 to english language
18. animals/
19. 17 not 18

PICO 8: Are patients on immunotherapy prior to liver transplantation at risk for rejection?

A review of PubMed was carried out until October 31, 2022. The search strategy used included:

1. "Immunotherapy" or "immune checkpoint inhibitors"
2. "Hepatocellular carcinoma"
3. "Liver transplantation"
4. "Neoadjuvant" or "bridge"

PICO 9: What is the best way to assess response to immunotherapy?

A review of PubMed was carried out until October 31, 2022. The search strategy used included:

1. "Immunotherapy" or "immune checkpoint inhibitors"
2. "Hepatocellular carcinoma"
3. "Pathologic" or "radiologic"
4. "Response"

PICO 10: What is the safety of the combined treatment with locoregional therapy and immunotherapy in the setting of transplantation?

MEDLINE and EMBASE were searched from 2012 to June 17, 2022. The search strategy used was:

1. Carcinoma, Hepatocellular/
2. (liver neoplasm$ or hepatic neoplasm$ or liver cancer$ or cancer of the liver or hepatocellular cancer$ or hepatocellular carcinoma$ or hepatic cancer$ or liver cell cancer$ or liver cell carcinoma$ or hepatoma$).ti,ab.
3. 1 or 2
4. Immunotherapy/
5. (immunotherap$ or checkpoint inhibitor$ or check?point inhibitor$).ti,ab.
6. Ipilimumab/
7. Nivolumab/

8. (ipilimumab or tremelimumab or nivolumab or pembrolizumab or camrelizumab or durvalumab or avelumab or atezolizumab or spartalizumab or cemiplimab or toripalimab).ti,ab.
9. or/4-8
10. Ablation Techniques/
11. Chemoembolization, Therapeutic/
12. (loco?regional therap$ or locoregional therap$ or ablation or chemoembolotherapy or chemoembolization or chemoembolisation or TACE or radioembolization or radioembolisation or intra?arterial microbrachytherapy or intraarterial microbrachytherapy or selective internal radiation therapy).ti,ab.
13. or/10-12
14. Adverse effects/
15. (adverse event$ or adverse effect$ or side effect$).ti,ab.
16. survival.ti,ab.
17. or/14-16
18. (combin$ or plus or simultaneous$).ti,ab.
19. 3 and 9 and 13 and 17 and 18
20. limit 19 to yr="2012 - Current"
21. limit 20 to english language
22. animals/
23. 21 not 22
